# Altered Fecal Microbiotas and Organic Acid Concentrations Indicate Possible Gut Dysbiosis in University Rugby Players: An Observational Study

**DOI:** 10.3390/microorganisms9081687

**Published:** 2021-08-09

**Authors:** So Morishima, Naoko Oda, Hiromi Ikeda, Tomohiro Segawa, Machi Oda, Takamitsu Tsukahara, Yasuharu Kawase, Tomohisa Takagi, Yuji Naito, Mami Fujibayashi, Ryo Inoue

**Affiliations:** 1 Laboratory of Animal Science, Department of Applied Biological Sciences, Faculty of Agriculture, Setsunan University, Nagaotoge-cho 45-1, Hirakata, Osaka 573-0101, Japan; so_mrsm@yahoo.co.jp (S.M.); hiromi.ikeda@setsunan.ac.jp (H.I.); machi.oda@edu.setsunan.ac.jp (M.O.); 2 Department of Food Science and Human Nutrition, Faculty of Agriculture, Setsunan University, I Nagaotoge-cho 45-1, Hirakata, Osaka 573-0101, Japan; naoko.oda@setsunan.ac.jp (N.O.); mami.fujibayashi@setsunan.ac.jp (M.F.); 3 Division of Physical and Health Education, Setsunan University, Ikedanaka-machi 17-8, Neyagawa, Osaka 572-8508, Japan; tomohiro.segawa@setsunan.ac.jp (T.S.); kawase@mpg.setsunan.ac.jp (Y.K.); 4 Kyoto Institute of Nutrition & Pathology, Ujitawara, Kyoto 610-0231, Japan; tsukahara@kyoto-inp.co.jp; 5 Department of Molecular Gastroenterology and Hepatology, Graduate School of Medical Science, Kyoto Prefectural University of Medicine, 465 Kajii-cho, Kamigyo-ku, Kyoto 602-8566, Japan; takatomo@koto.kpu-m.ac.jp; 6 Department of Human Immunology and Nutrition Science, Kyoto Prefectural University of Medicine, 465 Kajii-cho, Kamigyo-ku, Kyoto 602-8566, Japan; ynaito@koto.kpu-m.ac.jp

**Keywords:** gut dysbiosis, university rugby player, dietary fiber, microbiota, organic acid

## Abstract

Gut eubiosis is essential for the host’s health. In athletes, the gut microbiota can be altered by several factors, including diets. While eubiotic gut microbiota in elite rugby players has been reported, our survey found that university rugby players suffered from loose stools and frequent urgency to defecate. To establish the causes of the condition, the microbiota and the concentrations of organic acids in fecal samples of university male rugby players (URP) were analyzed and compared with those of age-matching, non-rugby playing males (control). Body mass indices were significantly (*p* < 0.05) different between groups. Chao1 index was significant (*p* < 0.05) lower in URP than in control. The relative abundances of phyla Firmicutes and Bacteroidetes were significantly (*p* < 0.05) higher and lower, respectively, in URP than in control. Potential pathobiont genera *Collinsella*, *Enterobacter,* and *Haemophilus* were significantly (*p* < 0.05) abundant, whereas beneficial *Akkermansia* was lower (*p* < 0.05) in URP than in control. Succinate, a potential causative of gut inflammation, was five-fold higher in URP than in controls. Our findings all but confirmed that the dysbiotic status of gut in URP.

## 1. Introduction

The gut microbiota has been intensively investigated in the last decade and, as a result, it is now clear that gut dysbiosis, which is caused by an abnormal composition of the gut microbiota and abnormal concentrations of their metabolites, can lead to the development of various gastrointestinal and systemic diseases [1,2,3,4]. Therefore, eubiosis is now regarded as essential to maintain the health of the host.

A growing body of evidence suggests that exercise can alter the composition of the gut microbiota and their metabolites. In general, simple, regular exercise can be beneficial for the gut microbiota. For example, increases in the abundance of butyrate-producing bacteria and the concentration of butyrate were observed in human feces following physical exercise [5]. Butyrate in the gut has been demonstrated to confer various health benefits to the host, including regulation of cell proliferation and differentiation and anti-inflammatory [6]. In addition, exercise has been shown to help increase the alpha-diversity in the gut microbiota of both animals and humans [7,8]. For example, Allen, et al. [9] reported that exercise-induced changes in the gut microbiota was likely associated with lower gut inflammation in a mouse model.

Paradoxically, some studies have reported potential adverse effects of exercise on the gut microbiota. For instance, Jang [10] reported that a reduced number of beneficial bacteria such as Bifidobacterium and butyrate-producing bacteria dwelt inside the gut of male bodybuilding athletes than in those not engaged in this physical activity. Recently, we also found that succinate, a metabolite of the gut microbiota that possibly causes gut inflammation and diarrhea, accumulated in the feces of female elite endurance runners when compared with age-matching but non-athlete females [11]. Those adverse exercise effects were likely caused by factors such as altered gut motility and transit, resulting from strenuous physical and psychological efforts [12,13]. In addition, site-specific oxidative stress and intestinal injury, resulting from exercise-induced repeated ischemia-reperfusion, are also likely factors leading to gut dysbiosis [14]. 

The composition of the diets of athletes may also cause gut dysbiosis. For example, athletes are recommended to consume high amounts of monosaccharides to maximize the storage of glycogen and sustain the level of blood glucose during exercise [15]. Moreover, to satisfy their muscle accretion needs, the ingestion of animal protein ingestion is high in athletes [10,16,17]. However, to prevent gastrointestinal disturbances, they can also be advised to lower their intake of dietary fiber [10,18]. According to Jang et al. [10], an unbalanced micronutrition, ensuing from a high-protein, low-dietary fiber diet, can decrease the number of beneficial bacteria in the gut microbiota of bodybuilders. 

In the case of elite professional rugby players, the ideal composition of their gut microbiotas should include a high diversity and abundance of beneficial bacteria such as *Roseburia* spp. [16]. Clarke et al. [16] reported that the levels of inflammation and metabolic markers in the blood of elite rugby players were equal to or better than those of healthy non-players. By contrast, metabolic markers such as low-density lipoprotein (LDL) cholesterol in blood, as well as the prevalence of non-alcoholic fatty liver disease (NAFLD) are higher in university rugby players than in that of control subjects [19]. 

A recent study reported gut dysbiosis as a potential cause of NAFLD [20]. In addition, a brief-type self-administered diet history questionnaire (BDHQ) to university rugby players performed in previous work found that the average dietary fiber intake was about 12 g/day, which is less than one-third that of the professional rugby players (39 g/day) [16]. Furthermore, the data showed that the feces of 76% (42/55) of university rugby players were loose (Bristol stool scale type 5, 6, or 7), suggesting a diarrheic pattern, and 78% (43/55) reported to have an urgency to defecate more than once per week. Based on this previous evidence, we hypothesized that the gut microbiotas of university rugby players might have been in a dysbiotic state. To confirm this hypothesis, we analyzed the microbiotas and the concentrations of organic acids in fecal samples from other university rugby players and those from age-matching, non-rugby playing males and compared the results.

## 2. Materials and Methods

### 2.1. Ethics Statements and Study Participants

This study was approved by the Ethical committee of Setsunan University and conducted as per their guidelines (approval number: 2020-037, approval date: 25 November 2020). Written informed consents were obtained from all participants. 

The enrollment of subjects and sample collection were conducted from December 2020 to February 2021. Eighty-eight male rugby players from Setsunan University were chosen and assigned to a group named *university rugby players* (URP). Of these players, 57 were forwards (FWs) and 31 backs (BKs). The requisites used to consider a participant for the URP group were as follows: (1) healthy male belonging to Setsunan university’s rugby team; (2) regularly participating in the team’s practices (at least 6 days per week, 3 h per day) and (3) with no previously diagnosed gastrointestinal disorders. Twenty healthy male students, also with no previously diagnosed gastrointestinal disorders, from the same university but not accustomed to exercise regularly, were recruited for the control group. Although higher BMIs were expected in URP (>25) than in controls based on previous studies [18,19], no BMI adjustment was conducted in the present study. The reason for this is “healthy” was a solid requisite for selecting participants, while participants not conducting regular exercise and had BMI higher than 25 are categorized as overweight or obese [21]. 

All participants provided fecal samples to the study, which were collected by themselves with ad-hoc scoop and container sets (Sarstedt K.K., Tokyo, Japan). The fecal samples were kept at 4 °C at all times and brought to our facilities within 24 h after collection. After reception, the fecal samples were stored at −20 °C or lower.

### 2.2. Analysis of the Fecal Microbiota 

Fecal DNA extraction, library preparation and deep sequencing using an MiSeq apparatus (Illumina K.K., Tokyo, Japan) were conducted as previously described [22]. Briefly, fecal DNA was extracted from 25 mg of feces by QuickGene DNA Tissue kit SII (KURABO, Osaka, Japan). The V3-4 region of 16S rRNA genes in each sample was amplified by primers 341F and 805R containing 5′ overhang adapter sequence for 2nd PCR. The amplicon was purified by NucleoFast 96 PCR plates (TaKaRa bio, Shiga, Japan) and a unique combination of dual indices (I5 and I7 index) was attached by 2nd PCR. After purification of the amplicons, concentration of each sample was normalized with a SequelPrep Normalization Plate Kit (Thermo Fisher, Tokyo, Japan) and pooled and concentrated by AMPure XP beads (Beckman Coulter, Tokyo, Japan). Ten pM of the library combined with 20% of phiX Control (Illumina) was sequenced with a 285-bp paired-end bases on the MiSeq using MiSeq Reagent Kit v3 (600 cycles; Illumina). 

Sequence data analysis was carried out as previously reported [23] with minor modifications. Demultiplex was performed by Quantitative Insights Into Microbial Ecology (QIIME ver.1.9.1) software [24]. Quality filtering, dereplication, singleton removal was performed using VSEARCH (ver. 2.15.1) [25], and clustering OTU and chimera check were conducted by USEARCH (ver.9.2.64) [26] and UCHIME2 [27], respectively. Taxonomy assignment of each OTU was carried out by RDP classifier (ver.2.2) [28] with the Greengenes database (13_8). Alpha and beta diversity analyses were conducted using R package “phyloseq (ver.1.30.0)” [29]. 

### 2.3. Analysis of the Concentrations of Fecal Organic Acids 

Ion-exclusion high-performance liquid chromatography was carried out to measure the concentrations of fecal organic acid, as per Tsukahara, et al. [30]. In brief, 0.3 g of feces was mixed with 0.6 mL of distilled water and the diluents was further mixed with 90 µL of 12% perchloric acid (*v*/*v*). After centrifugation (13,000× *g*, 4 °C, 10 min), the supernatants were filtered using a 0.45 μm cellulose acetate membrane filter (Cosmonice Filter W, Nakalai Tesque, Kyoto, Japan) and transferred to a vial. Then, it was injected into the high-performance liquid chromatography apparatus with an SIL-10 autoinjector (Shimadzu, Kyoto, Japan). Organic acids were separated by two serial organic acid columns (Shim-pack SCR-102H, Shimadzu) with a guard column (SCR-102HG; Shimadzu) at 45 °C with isocratic elution (0.8 mL/min) of 5 mmol/L ρ-toluene sulfonic acid aqueous solution using a solvent delivery pump (LC-10ADvp; Shimadzu) with an online degasser (DGU-12A; Shimadzu). Organic acids were detected with an electronic conductivity detector (Waters 431; Waters, Tokyo, Japan) after post-column dissociation (0.8 mL/min) with 5 mmol/L ρ-toluene sulfonic acid, 20 mmol/L bis-Tris and 100 μmol/L ethylenediaminetetraacetic acid using a solvent delivery pump (LC-10ADvp; Shimadzu). Organic acids were quantified with a system controller (CBM-20A; Shimadzu).

### 2.4. Statistical Analysis

In two group comparisons (FWs vs. BKs and URP vs. control), differences in alpha-diversity and the concentrations of fecal organic acids were evaluated by the Welch’s *t*-test. The relative abundances of bacterial genera were compared using the Wilcoxon rank sum test. The beta diversity of the fecal microbiota were compared with a permutational multivariate analysis of variance (PERMANOVA) by QIIME. All values are expressed as the means ± the standard deviations. Differences between the means were considered to be significant if *p* < 0.05. All statistical analyses were carried out using R (ver.4.0.2), unless otherwise specified.

## 3. Results

### 3.1. Corrections of the Experimental Groups

The fecal sample of one participant in the control group was excluded from the present study because diarrheic stool was submitted (met exclusion criteria of control subject). Hence, the number of participants in the control group totaled 19. Body mass indices were significantly different between groups (Table 1). Comparisons of the alpha- and beta-diversity in the fecal microbiota and the concentrations of organic acids did not find significant differences between the subgroups FWs and BKs (Supplement Table S1 and Supplement Figure S1). Therefore, as in the study by Clarke et al. [16], all rugby players were treated as one single group. 

### 3.2. Analysis of the Fecal Microbiota

**Figure 1:**
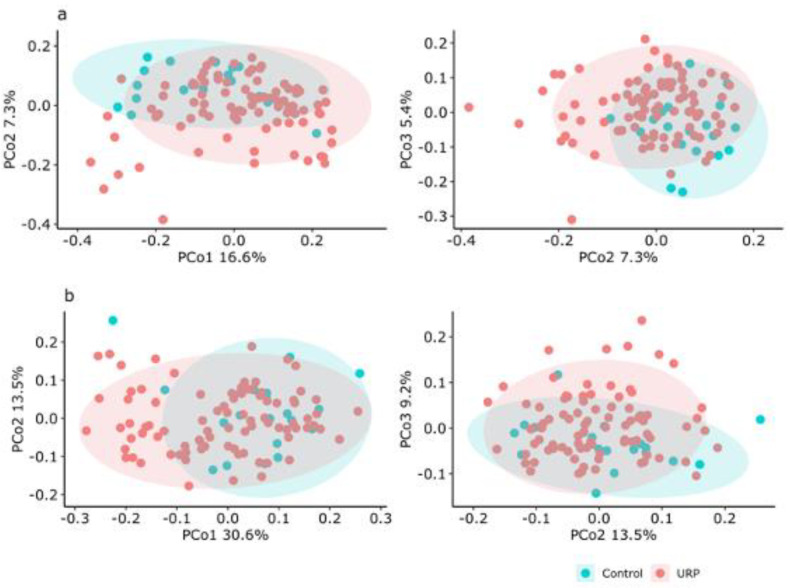
PCoA analysis of the fecal microbiota in fecal samples from the study participants, based on unweighted (**a**) and weighted (**b**) UniFrac distances. Ellipses enclosing clusters indicate 95% confidence interval. Significance (*p* < 0.05) was analyzed by PERMANOVA. Control: Age-matching, non-rugby playing males. URP: University rugby players.

The alpha-diversity analysis of the fecal microbiota showed a significant (*p* < 0.05) lower Chao1 index (richness) in the URP group (164.85 ± 47.84) than in the control group (206.74 ± 71.07) (Table 2), but no significant differences were found for the Shannon index (evenness) (Table 2). The beta-diversity based on both weighted and unweighted UniFrac distances were significantly different between the URP and control groups (Figure 1).

**Figure 2:**
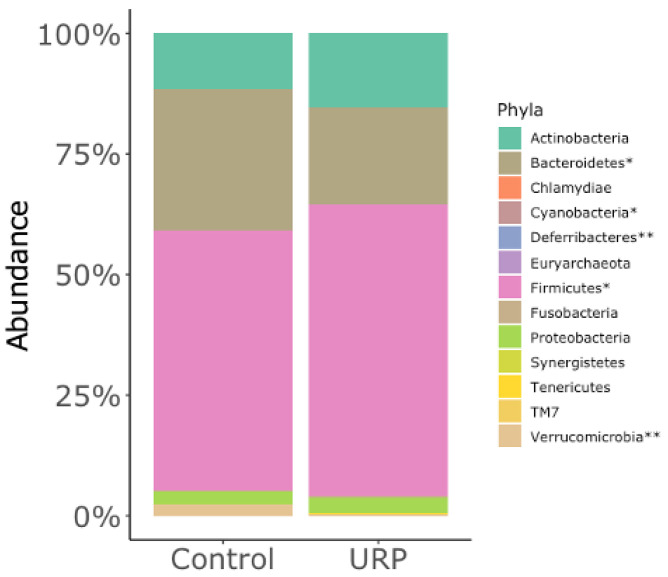
Relative abundances of fecal bacteria at phylum level. The asterisks indicate significance (* *p* < 0.05 and ** *p* < 0.01) in the relative abundances of phyla between groups.

In a taxonomic comparison, the relative abundances of 5 of 13 phyla were significantly different. In particular, phyla Firmicutes and Bacteroidetes were significantly higher and lower, respectively, in the URP group than in the control group (Figure 2). Among relatively abundant (>0.1%) bacteria, 17 bacterial genera were significant different between the URP and control groups at genus level (Table 3). For example, the relative abundance of genus *Bacteroides* was markedly lower (*p* < 0.05) in the URP group (14.77 ± 11.46) than in the control group (23.77 ± 11.92). In contrast, the relative abundance of *Collinsella* was more than 2-fold higher in the URP group (5.40 ± 5.99%) than in the control group (2.51 ± 3.06%). Similarly, *Megamonas* (4.70 ± 8.95%) and *Enterobacter* (0.43 ± 1.19%) were more than 10-fold higher in the URP group than in the control group (0.30 ± 1.30 and 0.02 ± 0.05%, respectively). The remaining bacterial genera whose relative abundances were significantly different between groups, and whose average relative abundances were lower than 0.1%, can be found in Supplementary Table S2.

### 3.3. Concentrations of Fecal Organic Acids

The concentrations of organic acids in the feces are shown in Table 4. The concentration of succinate was more than 5-fold higher in the URP group than in the control group (1.6 ± 3.1 vs. 0.3 ± 0.5). Nonetheless, the concentrations of branched chain fatty acids (BCFAs) isobutyrate (0.5 ± 1.5 vs. 3.0 ± 4.6) and isovalerate (<0.1 vs. 1.3 ± 1.0) were significantly lower in the URP group than in the control group.

## 4. Discussion

In the present study, we analyzed the microbiota and the concentrations of organic acids in the feces of 88 university male rugby players (51 FWs and 37 BKs) and 20 age-matching, non-rugby playing males. Our analyses found that there were distinct profiles between rugby players and controls.

Rugby football is a team sport in which players are divided into FWs and BKs. FWs typically have larger body masses than BKs and thus they ingest higher energy foods [19]. Physical exercise performed by players are also different, with FWs conducting short-duration, high anaerobic activity, while BKs perform more sprint and endurance running drills. Due to these physical and exercise differences, in the present study, it was expected the fecal microbiota and their metabolites concentrations to be different between player types. Surprisingly, no measurable differences in the fecal microbiota and the concentrations of organic acids were found between player types. Nirengi et al. [19] reported that the composition of macronutrients in male university rugby players and, after being normalized with the total energy, it did not differ between FWs and BKs. Therefore, it can be theorized that, as long as the compositions of macronutrients were similar, different physical exercises performed by FWs and BKs would have exerted little effect on the gut microbiota of the university rugby players studied. However, at the time of sample collection, training conducted by all rugby players in this study, regardless of positions, consisted of teamwork exercise for the league season. In other words, the exercises for the off-season are different to those for the league season, and conducted separately and according to positions. It is recommended that in future research, fecal samples be collected during both league and off-season and differences in the fecal gut microbiota be evaluated according to positions.

In the comparison between the URP and control groups and according to the Chao1 index, the richness of fecal microbiota was significantly lower in the URP group than in the control group, which contradicted the results from a previous study on elite rugby players [16].

Significant differences in the fecal microbiota compositions between the experimental groups were detected not only by beta-diversity analysis (based on UniFrac distances), but also by relative abundance analysis of specific genera. The most palpable differences were found in phylum Bacteroidetes and its lower taxonomic level genus *Bacteroides*. Indeed, as in the work by Clark et al. [16], the relative abundances of both Bacteroidetes and *Bacteroides* in the present study were significantly lower in the URP group than in the control group. Hence, we believe low relative abundances of Bacteroidetes and *Bacteroides* are characteristic features in the gut microbiota of male rugby players, elite or otherwise. 

In the present work, the relative abundance of *Megamonas* was also different in the fecal microbiota of URP and control groups. A relative abundance of *Megamonas* is positively correlated with serum testosterone level in men [31], and the level of serum testosterone is high in trained men, especially in those performing resistance exercises [32,33]. It remains unclear, nevertheless, whether *Megamonas* induces an increase in the level of serum testosterone or vice versa, but as far as the present study goes, it is likely that an increase in *Megamonas* in the URP group could be consequential to the sort of resistance exercises that rugby players regularly do.

Differences in four bacterial genera in the fecal microbiota of the URP group suggested a dysbiotic status of the gut. *Collinsella*, *Enterobacter* and *Haemophilus* were found significantly abundant, whereas *Akkermansia* was significantly less abundant in the URP group than in the control group. In a preprint released online by Medawar et al. [34], *Collinsella* was suggested as an inversely health-related genus. In addition, *Collinsella* has been observed abundantly in the gut of a host having a low intake of dietary fiber [35,36]. Thus, in the present work, it is very possible that a high abundance of *Collinsella* was associated with a low intake of dietary fiber by the university rugby players, and possibly with the eventful recurrence of diarrhea. As for *Enterobacter* and *Haemophilus*, they are reported to be abundant in the microbiota of Crohn’s disease patients [37]. In contrast, *Akkermansia* is known to be a beneficial bacterium [38]. Although *Akkermansia* was found by Clark et al. [16] to be higher in elite rugby players, in the URP group of the present work, it only comprised one-fifth of that in control group.

The concentrations of organic acids found in the feces of the URP group also indicated a dysbiotic gut microbiota. For example, succinate was notably higher in the URP group than in the control group. Accumulation of succinate putatively increases the osmotic pressure in the lumen and reduces water absorption rate in the intestine, resulting in loose or diarrheal stools [39,40]. Moreover, luminal succinate accumulation has been suggested to be associated with gut inflammation in humans [41]. With regard to BCFAs, the concentrations of isobutyrate and isovalerate inversely correlate with fecal water content in adult men [42]. Thus, this evidence conformably fits with the incidence of loose stools in the URP group. No differences were found between the concentration of butyrate in the feces of the experimental groups. However, when the concentration of butyrate was analyzed at individual level in the URP group, 22 of 87 players had it below detection, which was not the case in the control group (*p* = 0.01 by the Fisher’s exact test). Butyrate, a major short-chain fatty acid produced by microbiota in the human gut [6], has been reported to be high in elite rugby players [43]. Thus, this result also seemed to confirm the dysbiotic status of the gut microbiota in the URP group. 

It must be mentioned that the present study had limitations. For example, the backgrounds of participants were not well controlled. This was an observational study, and the highest priority was placed on recruiting participants meeting the minimum requirements for each experimental group. Therefore, parameters possibly affecting the gut environment, such as alcohol, antibiotic consumption and pre/probiotic consumption were not listed in the excluding criteria. For a similar reason, evaluation of dietary habits was beyond the scope of this study. Our previous study [19] that evaluation of dietary habits became a burden to participants and discouraged them from participating. 

## 5. Conclusions

In conclusion, in the present work, the low richness and high relative abundances of possible pathobiont bacterial genera in the microbiota of university male rugby players, along with abnormal concentrations of organic acids in their fecal samples, seemed to imply a dysbiotic status of their gut microbiota. Taken together, the gathered data seemed to be self-explanatory of the fact that university rugby players suffered from constant loose stool and urgency to defecate above average. No evaluation of the nutritional intake in subjects enrolled in the present study was carried out. However, based on daily calorie intake, the dietary fiber intake by URP was probably lower than that of controls. This conjecture seems to be confirmed by the high abundance of *Collinsella* in the feces of URP, implying the nonexistence of eubiosis. To better define the relationship between exercise, dietary fiber and the gut environment, well-controlled background and nutritional intake evaluations of participants are recommended for future studies. An ongoing study at this laboratory is investigating the effect of dietary intervention, including the use of prebiotics, on the dysbiotic guts of university male rugby players.

## Figures and Tables

**Table 1:** Ages and body mass indices of participants in the present study.

		URPs ^†^	
	Controls	FWs	BKs
Age	20.1 ± 1.8	20.5 ± 1.2	20.8 ± 1.3
BMI	20.8 ± 2.6 *^a^	30.7 ± 3.6 ^b^	26.1 ± 1.8 ^c^

URP: University rugby players. Team positions: FWs, forwards.; BKs, backs. Controls: Age-matching, non-rugby playing males. BMI: Body mass indices. All subjects were non-smokers. * One of the subjects in control declined reporting his BMI but confirmed his BMI was lower than 25. ^†^ Two of UPRs were experienced mild concussion within one month prior to sample collection. Different letters (^a–c^) indicate significance (*p* < 0.05).

**Table 2:** Alpha diversity indices of the microbiota in fecal samples from the control and URP groups.

Index	Controls	URP	*p*-Value
Chao1	206.74 ± 71.07	164.85 ± 47.84	0.02 *
Shannon	4.75 ± 0.55	4.53 ± 0.52	0.13

Controls: Age-matching, non-rugby playing males. URP: University rugby players. Significance (* *p* < 0.05) was calculated using the Welch’s *t*-test.

**Table 3:** Comparison of fecal bacterial genera in the fecal samples from the control and URP groups.

Taxonomy			Control	URP	*p*-Value
Order	Family	Genus			
**Higher in URP**					
Clostridiales	*Veillonellaceae*	*Megamonas*	0.299 ± 1.303	4.698 ± 8.953	<0.001 **
Coriobacteriales	*Coriobacteriaceae*	*Collinsella*	2.512 ± 3.064	5.404 ± 5.989	0.025 *
Pasteurellales	*Pasteurellaceae*	*Haemophilus*	0.157 ± 0.475	0.795 ± 2.305	0.004 **
Clostridiales	*Clostridiaceae*	*SMB53*	0.125 ± 0.124	0.730 ± 1.934	0.010 *
Enterobacteriales	*Enterobacteriaceae*	*Enterobacter*	0.020 ± 0.045	0.433 ± 1.193	0.002 **
Fusobacteriales	*Fusobacteriaceae*	Unclassified	0.001 ± 0.005	0.287 ± 0.924	0.028 *
Actinomycetales	*Actinomycetaceae*	*Actinomyces*	0.076 ± 0.108	0.164 ± 0.198	0.019 *
**Lower in URP**					
Clostridiales	*Lachnospiraceae*	*Clostridium*	0.587 ± 0.645	0.521 ± 1.547	0.013 *
Clostridiales	*Ruminococcaceae*	Unclassified	0.178 ± 0.275	0.107 ± 0.304	0.012 *
Coriobacteriales	*Coriobacteriaceae*	*Eggerthella*	0.150 ± 0.153	0.075 ± 0.090	0.027 *
Bacteroidales	*[Odoribacteraceae]*	*Odoribacter*	0.200 ± 0.274	0.053 ± 0.140	0.001 **
Desulfovibrionales	*Desulfovibrionaceae*	*Bilophila*	0.241 ± 0.262	0.053 ± 0.093	0.001 **
Clostridiales	Unclassified	Unclassified	0.593 ± 1.249	0.318 ± 0.924	0.008 **
Bacteroidales	*Rikenellaceae*	Unclassified	1.283 ± 1.328	0.406 ± 1.128	<0.001 **
Clostridiales	*Ruminococcaceae*	*Oscillospira*	1.978 ± 1.478	0.938 ± 1.277	<0.001 **
Verrucomicrobiales	*Verrucomicrobiaceae*	*Akkermansia*	2.322 ± 5.611	0.436 ± 2.347	0.010 **
Bacteroidales	*Bacteroidaceae*	*Bacteroides*	23.771 ± 11.922	14.765 ± 11.455	0.004 **

Genera with relative abundances higher than 0.1% and having significant differences between groups are listed. Significance (* *p* < 0.05 and ** *p* < 0.01) was analyzed using the Wilcoxon rank sum test. Genera with relative abundances lower than 0.1% and having significant differences between groups are listed in Table S2.

**Table 4:** Concentrations of organic acids in fecal samples from the control and URP groups.

Organic Acids	Controls	URP	*p*-Value
(mmol/kg Wet Feces)			
Succinate	0.3 ± 0.5	1.6 ± 3.1	<0.001 **
Lactate	0.2 ± 0.6	0.0 ± 0.1	0.27
Formiate	0.2 ± 0.4	0.3 ± 1.2	0.58
Acetate	35.3 ± 18.2	38.5 ± 20.2	0.51
Propionate	11.6 ± 5.2	10.4 ± 8.5	0.44
*iso*Butyrate	3.0 ± 4.6	0.5 ± 1.5	0.03 *
*n*Butyrate	7.4 ± 6.7	5.2 ± 4.6	0.19
*iso*Valerate	1.3 ± 1.0	0.0 ± 0.2	<0.001 **
*n*Valerate	<0.1	0.0 ± 0.2	0.32

Controls: Age-matching, non-rugby playing males. URP: University rugby players. Significance (* *p* < 0.05, ** *p* < 0.01) was calculated using the Welch’s *t*-test.

## Data Availability

The sequence data have been deposited in the DDBJ Sequence Read Archive (DRA) under accession number DRA012258 (available from 1 August 2021).

## Supplementary Materials

The following are available online at https://www.mdpi.com/article/10.3390/microorganisms9081687/s1, Figure S1: PCoA analysis of the microbiotas in fecal samples from university rugby players, based on unweighted and weighted UniFrac distances. Ellipses enclosing clusters indicate 95% confidence interval. Significance was analyzed by PERMANOVA. Team positions: FWs, Forwards; BKs, Backs, Table S1: Alpha diversity indices of the microbiotas in fecal samples from university rugby players. Table S2: Bacterial genera that differed in fecal samples of the control and URP groups.
