# Supplementary material for: Altered Fecal Microbiotas and Organic Acid Concentrations Indicate Possible Gut Dysbiosis in University Rugby Players: An Observational Study
Source: Microorganisms. 2021 Aug 9;9(8):1687. doi: 10.3390/microorganisms9081687 (PMC8400784; doi:10.3390/microorganisms9081687)
Supplement: Supplementary file 1 [file microorganisms-09-01687-s001.zip › microorganisms-1284560.pdf]

Supplementary Table S1. Alpha diversity indices of the microbiotas in fecal samples from university rugby players

Index	FWs			BKs			*P*-value
Chao1	161.18	±	47.78	169.91	±	48.11	0.36
Shannon	4.54	±	0.50	4.52	±	0.55	0.79

Team positions: FWs, forwards.; BKs, backs

Significance (*P<0.05 and **P<0.01) was analyzed using the Welch's t-test.

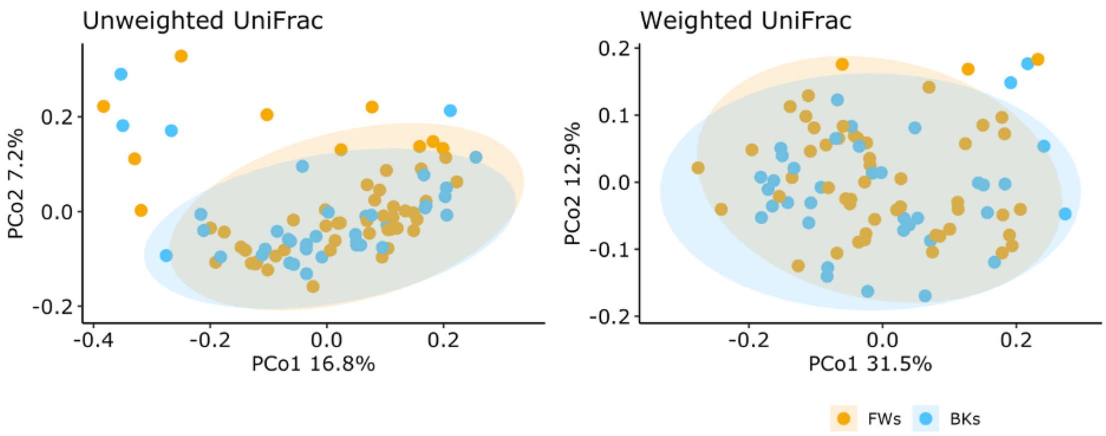

Supplementary Figure S1. PCoA analysis of the microbiotas in fecal samples from university rugby players, based on unweighted and weighted UniFrac distances. Ellipses enclosing clusters indicate 95% confidence interval. Significance was analyzed by PERMANOVA. Team positions: FWs, Forwards; BKs, Backs

Supplementary Table S2. Bacterial genera that differed in fecal samples of the control and URP groups (average abundance < 0.1%)

Taxonomy			Control			URP			*P*-value	
Order	Family	Genus								
Clostridiales	*Lachnospiraceae*	Unclassified	0.092	±	0.132	0.083	±	0.243	0.028	*
Erysipelotrichales	*Erysipelotrichaceae*	*Coprobacillus*	0.097	±	0.167	0.051	±	0.194	0.003	**
Bacteroidales	S24-7	Unclassified	0.034	±	0.077	0.102	±	0.677	<0.001	**
Clostridiales	*Christensenellaceae*	Unclassified	0.082	±	0.239	0.039	±	0.235	0.001	**
Clostridiales	Unclassified	Unclassified	0.022	±	0.044	0.073	±	0.103	0.028	*
Bacillales	*Bacillaceae*	*Bacillus*	0.004	±	0.009	0.080	±	0.158	0.029	*
Clostridiales	*Lachnospiraceae*	*Defluviitalea*	0.065	±	0.096	0.016	±	0.042	0.003	**
Pasteurellales	*Pasteurellaceae*	*Aggregatibacter*	0.002	±	0.007	0.062	±	0.207	0.016	*
Enterobacteriales	*Enterobacteriaceae*	*Citrobacter*	0.004	±	0.014	0.057	±	0.217	0.021	*
Lactobacillales	*Streptococcaceae*	*Lactococcus*	0.003	±	0.011	0.053	±	0.300	0.006	**
Actinomycetales	*Micrococcaceae*	*Rothia*	0.012	±	0.023	0.044	±	0.089	0.005	**
Clostridiales	*Ruminococcaceae*	*Anaerotruncus*	0.040	±	0.062	0.008	±	0.019	<0.001	**
Lactobacillales	*Carnobacteriaceae*	*Granulicatella*	0.007	±	0.006	0.030	±	0.054	0.007	**
Lactobacillales	*Lactobacillaceae*	*Pediococcus*	0.015	±	0.060	0.019	±	0.164	0.040	*
Erysipelotrichales	*Erysipelotrichaceae*	cc_115	0.010	±	0.017	0.002	±	0.005	<0.001	**
Bacteroidales	*Rikenellaceae*	*Rikenella*	0.010	±	0.031		<0.001		0.002	**
Streptophyta	Unclassified	Unclassified	0.001	±	0.002	0.007	±	0.025	0.033	*
Clostridiales	*Peptococcaceae*	rc4-4	0.005	±	0.014	0.003	±	0.019	0.038	*
Clostridiales	*Christensenellaceae*	*Christensenella*	0.006	±	0.012	0.001	±	0.004	0.005	**
Clostridiales	*Christensenellaceae*	Unclassified	0.005	±	0.010	0.001	±	0.003	0.001	**
Clostridiales	*Eubacteriaceae*	*Anaerofustis*	0.003	±	0.004	0.001	±	0.004	0.005	**
Clostridiales	*Dehalobacteriaceae*	*Dehalobacterium*	0.003	±	0.009	0.001	±	0.006	0.030	*
Actinomycetales	*Corynebacteriaceae*	*Corynebacterium*	0.003	±	0.009	0.001	±	0.009	0.036	*
Pseudomonadales	*Moraxellaceae*	*Acinetobacter*	0.003	±	0.006		<0.001		<0.001	**
Sphingomonadales	*Sphingomonadaceae*	*Sphingomonas*		<0.001		0.003	±	0.004	<0.001	**
Bacteroidales	*Porphyromonadaceae*	*Dysgonomonas*	0.002	±	0.007		<0.001		0.033	*
Deferribacterales	*Deferribacteraceae*	*Mucispirillum*	0.001	±	0.003		<0.001		<0.001	**
Clostridiales	[*Tissierellaceae*]	WAL_1855D	0.001	±	0.002	0.000	±	0.003	<0.001	**
Campylobacterales	*Helicobacteraceae*	*Flexispira*		<0.001		0.001	±	0.002	0.023	*
Campylobacterales	*Helicobacteraceae*	Unclassified		<0.001		0.001	±	0.002	0.047	*
Bacteroidales	Unclassified	Unclassified	0.000	±	0.002		<0.001		0.033	*
Pseudomonadales	*Moraxellaceae*	*Enhydrobacter*	0.000	±	0.001		<0.001		0.033	*

Genera with relative abundances lower than 0.1% and having significant differences between groups are listed. Significance (*P<0.05 and **P<0.01) was analyzed using the Wilcoxon rank sum test.
